# Oxidized LDL Modify the Human Adipocyte Phenotype to an Insulin Resistant, Proinflamatory and Proapoptotic Profile

**DOI:** 10.3390/biom10040534

**Published:** 2020-04-01

**Authors:** Concepción Santiago-Fernández, Flores Martin-Reyes, Mónica Tome, Luis Ocaña-Wilhelmi, Jose Rivas-Becerra, Franz Tatzber, Edith Pursch, Francisco J. Tinahones, Eduardo García-Fuentes, Lourdes Garrido-Sánchez

**Affiliations:** 1 Unidad de Gestión Clínica de Aparato Digestivo, Hospital Universitario Virgen de la Victoria, 29010 Malaga, Spain; conchisantiagofernandez@gmail.com; 2 Instituto de Investigación Biomédica de Málaga-IBIMA, 29010 Malaga, Spain; floresmarey@hotmail.com (F.M.-R.); lourgarrido@gmail.com (L.G.-S.); 3 Universidad de Málaga, 29010 Malaga, Spain; 4 Unidad de Gestión Clínica de Endocrinología y Nutrición, Hospital Universitario Virgen de la Victoria, 29010 Malaga, Spain; 5 Unidad de Gestión Clínica de Endocrinología y Nutrición, Hospital Regional Universitario, 29010 Malaga, Spain; doctoratome@gmail.com; 6 Unidad de Gestión Clínica de Cirugía General, Digestiva y Trasplantes, Hospital Universitario Virgen de la Victoria, 29010 Malaga, Spain; luisowilhelmi@hotmail.com; 7 Unidad de Gestión Clínica de Cirugía General, Digestiva y Trasplantes, Hospital Regional Universitario, 29010 Malaga, Spain; doctopep@hotmail.com; 8 Medical University of Graz, Centre of Molecular Medicine, Institute of Pathophysiology & Immunology, 8010 Graz, Austria; franz@tatzber.at; 9 University of Applied Sciences Technikum Wien, Institute of Biochemistry, 1200 Vienna, Austria; werner.pursch@aon.at; 10 CIBER Fisiopatología Obesidad y Nutrición (CIBERObn), Instituto Salud Carlos III, 29010 Malaga, Spain

**Keywords:** oxidized low-density lipoprotein, scavenger receptors, adipocyte, inflammation, apoptosis

## Abstract

Little information exists in humans on the regulation that oxidized low-density lipoprotein (oxLDL) exerts on adipocyte metabolism, which is associated with obesity and type 2 diabetes. The aim was to analyze the oxLDL effects on adipocytokine secretion and scavenger receptors (SRs) and cell death markers in human visceral adipocytes. Human differentiated adipocytes from visceral adipose tissue from non-obese and morbidly obese subjects were incubated with increasing oxLDL concentrations. mRNA expression of SRs, markers of apoptosis and autophagy, secretion of adipocytokines, and glucose uptake were analyzed. In non-obese and in morbidly obese subjects, oxLDL produced a decrease in insulin-induced glucose uptake, a significant dose-dependent increase in tumor necrosis factor-α (TNF-α), IL-6, and adiponectin secretion, and a decrease in leptin secretion. OxLDL produced a significant increase of *Lox-1* and a decrease in *Cxcl16* and *Cl-p1* expression. The expression of *Bnip3* (marker of apoptosis, necrosis and autophagy) was significantly increased and *Bcl2* (antiapoptotic marker) was decreased. OxLDL could sensitize adipocytes to a lower insulin-induced glucose uptake, a more proinflammatory phenotype, and could modify the gene expression involved in apoptosis, autophagy, necrosis, and mitophagy. OxLDL can upregulate *Lox-1*, and this could lead to a possible amplification of proinflammatory and proapoptotic effects of oxLDL.

## 1. Introduction

Obesity is a risk factor of diabetes mellitus type 2 (T2DM) and atherosclerosis. The development of these pathologies is associated with a dysregulated secretion of adipocytokines, which is associated with the chronic low-grade inflammation observed in obesity [1]. In the regulation of adipocytokine secretion, several factors are involved such as different lipoproteins [2]. Oxidized low-density lipoproteins (oxLDL) play an important role in the evolution of obesity [3], metabolic syndrome [4], and cardiovascular disease [5]. Various studies suggest that adipocytes could be involved in the regulation of the metabolism of lipoproteins such as oxLDL. These oxLDL are uptaken by cells by means of scavenger receptors (SRs), which have been found in adipocyte cell lines such as 3T3-L1 in human and animal adipocytes [6]. Class A scavenger receptors (macrophage scavenger receptor 1 (MSR1) and macrophage receptor with collagenous structure (MARCO)), class B receptors (scavenger receptor class B, member 1 (SR-B1) and CD36), class E receptors (oxidized low density lipoprotein receptor 1 (LOX-1)), class F receptors (scavenger receptor class F member 1/2 (SRECI/II)), and class G receptors (C-X-C motif chemokine ligand 16 (CXCL16)) account for over 90% of oxLDL uptake [7,8]. As it is known, CD36 is an SR whose expression is regulated by oxLDL [9]. However, the other SRs can also have the ability to bind to oxLDL and may be involved in mediating the cellular effects of these oxLDL. Some toxic oxidized lipids (oxysterols, lipid peroxides, etc.) of oxLDL may stimulate the liver X receptor (LXR) pathway and the subsequent induction of LXR-target genes such as ATP-binding cassette transporters A1 (ABCA1) [10], and may lead to lysosomal damage, which may participate in apoptosis, cellular autolysis, and death [11,12]. Apoptosis induced by high concentrations of oxLDL is initiated by the decrease of antiapoptotic proteins (BCL2) and the increase and activation of caspase cascade (caspase 3 (CASP3)) [13]. Autophagy is a pathway mediated by lysosomes that degrades cytosolic components. OxLDL promotes apoptosis and autophagy in different cell types, but there is little information about these effects in human adipocytes. Furthermore, oxLDL produces a decrease in insulin sensitivity, possibly by inhibiting the signaling kinases responsible for the cellular response to insulin and/or by activating the nuclear factor kappa B subunit 1 (NF-κB) complex, which regulates genes involved in inflammation and cell survival [6].

The internalization of oxLDL by adipocytes could be involved in the clearance of oxLDL from plasma. However, its uptake by adipocytes may also be associated with an alteration of adipocyte metabolism and more specifically with the chronic low-grade inflammation associated with obesity. One study in 3T3-L1 demonstrated that oxLDL is endocytosed via CD36 [14]. However, most of the effects of oxLDL have been identified in macrophages, endothelial cells, or in 3T3-L1 adipocytes, but their effect on human adipocytes is not well known. 

Based on the evidence mentioned above, the aims of this study were to demonstrate the effects of oxLDL on different SRs, markers of inflammation, apoptosis, autophagy, and transcription factors involved in the regulation of oxLDL effects in adipocytes from non-obese and morbidly obese subjects. On the other hand, we wanted to evaluate the effect of oxLDL on the uptake of glucose by adipocytes and analyze the role of oxLDL in the development of insulin resistance.

## 2. Materials and Methods

### 2.1. Subjects

We evaluated 10 morbidly obese subjects who underwent biliopancreatic diversion of Scopinaro (BPD) at the Virgen de la Victoria University Hospital, Malaga (Spain) [15] and 10 non-obese subjects who underwent laparoscopic surgery for cholelithiasis at the Regional University Hospital, Malaga (Spain). Samples from these subjects were used to perform the experiments shown below. Table 1 summarizes the biochemical and anthropometric variables of the non-obese and morbidly obese subjects. The non-obese subjects were selected with a similar body mass index (BMI) to the average BMI of the population of our area (27.5 ± 2.1 Kg/m^2^) [16]. In addition, these non-obese subjects had a slightly high homeostasis model assessment of insulin resistance (HOMA-IR). These subjects were selected so that there were no significant differences between the non-obese and morbidly obese groups and that obesity was the only main factor to consider. We used a distribution of males/females similar to the distribution of male/female found in subjects who underwent bariatric surgery (1/2). Subjects were excluded if they were receiving insulin or hypoglycemic agents, had cardiovascular disease, arthritis, acute inflammatory disease, or infectious disease. All subjects were of Caucasian origin. The study was conducted in accordance with the guidelines laid down in the Declaration of Helsinki. All participants gave their written informed consent and the study was reviewed and approved by the Malaga Provincial Research Ethics Committee (CEI_CP13-00188).

### 2.2. Laboratory Measurements

Blood samples from all subjects before surgery were collected after a 10-h fast. The serum was separated and immediately frozen at −80 °C. Serum biochemical variables were measured in duplicate as previously described [15]. HOMA-IR was calculated: HOMA-IR = fasting insulin (μIU/mL) × fasting glucose (mol/L)/22.5.

### 2.3. Isolation of Stromal Vascular Fraction and Mature Adipocytes

The biopsy samples were washed in physiological saline and immediately processed. Visceral adipose tissues (VAT) from non-obese and morbidly obese subjects were digested with 3 mg/mL collagenase type II (Worthington Biochemical Corporation, Lakewood, NJ, USA) in Dulbecco’s Modified Eagle Medium (DMEM) for 45 min at 37 °C in a shaking water bath, as previously described [17]. Digests were filtered through a 100-μm cup filter. Adipocytes and stromal vascular fraction (SVF) were isolated by centrifugation (100 g and 3 min). The adipocytes were isolated and immediately frozen in QIAzol Lysis Reagent (Qiagen, GmbH, Hilden, Germany) at −80 °C until mRNA analysis. The pellet of the digested VAT samples contained the SVF.

### 2.4. In Vitro Differentiated Adipocyte Culture

**Figure 1:**
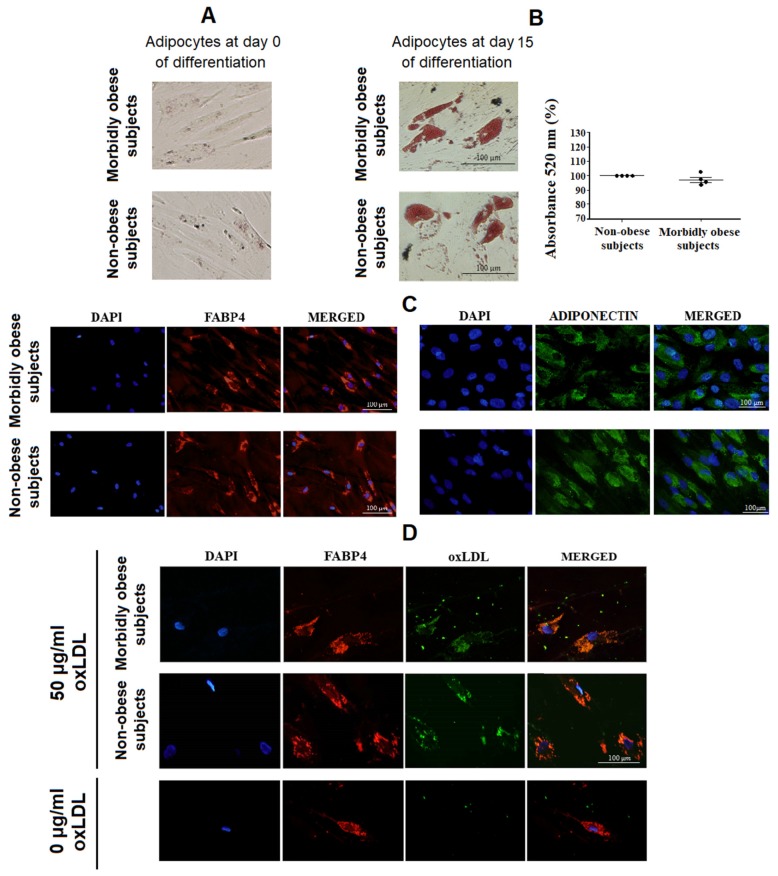
(**A**) Oil red-O staining of human mesenchymal stem cells at day 0 of differentiation; (**B**) Oil red-O staining and quantification in human visceral in vitro differentiated adipocytes after 15 days of differentiation; (**C**) immunofluorescence staining of FABP4 and adiponectin in human visceral in vitro differentiated adipocytes after 15 days of differentiation; (**D**) OxLDL uptake by visceral in vitro differentiated adipocytes. In vitro differentiated adipocytes were incubated without oxLDL (0 μg/mL oxLDL or negative control) and with 50 μg/mL oxLDL for 4 h.

All reagents were from Sigma-Aldrich (Sigma-Aldrich, St. Louis, MO, USA) unless otherwise specified. In vitro cultures were performed as previously described [17]. The pellet of SVF was washed twice with DMEM and treated with an erythrocyte lysis buffer for 10 min at room temperature. SVF were plated in DMEM supplemented with 20% fetal bovine serum (FBS), 1% L-glutamine, and 1% penicillin/streptomycin under standard culture conditions at 37 °C in a humidified atmosphere containing 5% CO_2_. The plating medium was changed every three days until confluence (80–90%). Newly confluent cultures were sub-cultured in six-well plates and grown to confluence. At this stage, designated as day 0 (Figure 1A), differentiation was induced by treatment with adipogenic medium composed of StemXVivo™ Osteogenic/Adipogenic Base Media with StemXVivo Adipogenic Supplement (R&D Systems, Inc., Minneapolis, MN, USA) for the differentiation of human mesenchymal stem cells (HMSC) into adipocytes. This medium was changed every three days. After 15 days of differentiation, adipocytes were differentiated [17,18] and presented a phenotype of mature adipocytes, as detected by Oil Red-O staining (Figure 1B) and fatty acid binding protein 4 (FABP4) (Figure 1C) and adiponectin (Figure 1C) immunofluorescence staining, markers of mature adipocytes [19]. The culture medium of in vitro differentiated adipocytes was changed, and adipocytes were incubated with 0, 25, and 50 μg/mL of malondialdehyde modified human LDL (oxLDL) (0, 25, and 50 ug protein/mL of malondialdehyde modified oxLDL) (MyBioSource, Inc., San Diego, CA, USA). We selected these oxLDL concentrations based on previous studies [20,21]. After 24 h of incubation with oxLDL, adipocytes were harvested and frozen in QIAzol Lysis Reagent (Qiagen, GmbH, Germany) at −80 °C until analysis. The culture medium was also collected, centrifuged at 300 g for 10 min, and frozen at −80 °C until analysis. Each treatment was performed in triplicate. The SVF used for differentiation into adipocytes were from individual subjects, not pooled.

### 2.5. Analysis of the Culture Medium

HMSC from non-obese and morbidly obese subjects were differentiated into adipocytes as described above. Human TNF-α, IL-6, leptin, and adiponectin levels were measured in the culture medium of the in vitro differentiated adipocytes incubated with 0, 25, and 50 μg/mL of oxLDL for 24h by enzyme-linked immunosorbent assay kits (R&D Systems) according to the manufacturer’s instructions. Data are expressed as mean ± standard error of the cytokine concentration per 10^6^ cells at the time of harvest.

### 2.6. RNA Extraction and Real-Time Quantitative PCR

Total RNA from mature adipocytes and in vitro differentiated adipocytes were isolated by RNeasy Lipid Tissue Mini Kit (Qiagen, GmbH, Germany) as previously described [15]. Total RNA was reverse transcribed using random hexamers as primers and transcriptor reverse transcriptase (Roche, Mannheim, Germany). The expression of genes was assessed by real-time PCR using an Applied Biosystems 7500 Fast Real-Time polymerase chain reaction System (Applied Biosystems, Darmstadt, Germany). Reactions were carried out in duplicate for all genes using specific TaqMan^®^ Gene Expression Assays: macrophage scavenger receptor 1 (*Msr1*) (Hs00234007_m1, RefSeq. NM_002445.3, NM_138715.2, NM_138716.2), chemokine (C-X-C motif) ligand 16 (*Cxcl16*) (Hs00222859_m1, RefSeq. NM_001100812.1, NM_022059.2), oxidized low density lipoprotein (lectin-like) receptor 1 (*Lox-1*) (Hs01552593_m1, RefSeq. NM_001172632.1, NM_001172633.1, NM_002543.3), collectin sub-family member 12 (*Cl-p1*) (Hs00560477_m1, RefSeq. NM_130386.2), *CD36* (Hs00169627_m1, RefSeq. NM_000072.3, NM_001001547.2, NM_001001548.2, NM_001127443.1, NM_001127444.1, NM_001289909.1, NM_001289911.1), B-cell lymphoma 2 (*Bcl2*) (Hs00153350_m1; RefSeq: NM_000633.2), caspase 3 (*Casp3*) (Hs00234387_m1; RefSeq: NM_004346.3, NM_032991.2), autophagy related 3 (*Atg3*) (Hs00223937_m1; RefSeq:NM_001278712.1, NM_022488.4), BCL2 interacting protein 3 (*Bnip3*) (Hs00969291_m1; RefSeq: NM_004052.3), nuclear factor, erythroid 2 like 2 (*Nrf2*) (Hs00975961_g1; RefSeq: NM_001145412.3, NM_001145413.3, NM_001313900.1, NM_001313901.1, NM_001313902.1, NM_001313903.1, NM_001313904.1, NM_006164.4), nuclear factor kappa B subunit 1 (*Nf-κB*) (Hs00765730_m1; RefSeq: NM_001165412.1, NM_001319226.1, NM_003998.3), nuclear receptor subfamily 1 group H member 3 (*NR1H3* or *Lxrα*) (Hs00172885_m1; RefSeq: NM_001130101.2, NM_001130102.2, NM_001251934.1, NM_001251935.1 and NM_005693.3), ATP binding cassette subfamily A member 1 (*Abca1*) (Hs01059137_m1; RefSeq: NM_005502.3), interleukin-6 (IL-6) (Hs00174131_m1; RefSeq: NM_000600.3), tumor necrosis factor-alpha (*Tnfα*) (Hs00174128_m1; RefSeq: NM_000594.3), C–C motif chemokine ligand 2 (*Ccl2* or *Mcp1*) (Hs00234140_m1; RefSeq: NM_002982.3) and insulin receptor (*Insr*) (Hs00961557_m1; RefSeq: NM_000208.3, NM_001079817.2). The threshold cycle (Ct) value for each sample was normalized with the expression of cyclophilin A (4326316E, RefSeq. NM_021130.3). SDS software 2.3 and RQ Manager 1.2 (Applied Biosystems, Foster City, CA, USA) were used to analyze the results with the comparative Ct method (2^−ΔCt^). In differentiated adipocyte cultures, results are shown as a percentage with regard to non-obese group without oxLDL (100%).

### 2.7. Immunohistochemical Staining

HMSC from non-obese and morbidly obese subjects were differentiated into adipocytes as described above. In vitro differentiated adipocytes were incubated with 50 μg/mL oxLDL for 4 h. After this incubation, cells were washed twice with phosphate-buffered saline (PBS). Adipocytes were fixed with 4% paraformaldehyde solution for 20 min at room temperature and with gentle agitation, and were washed twice with PBS. Subsequently, cells were blocked with triton in PBS + bovine serum albumin (BSA) 1%. Afterwards, cells were incubated with a monoclonal antibody to MDA-LDL (MDA-Apo-B) conjugated with FITC (MBS465035, MyBioSource, Inc., San Diego, CA, USA) diluted to 1/1000 for 2 h and a rabbit anti-FABP4 polyclonal antibody, Alexa Fluor^®^ 555 conjugated (bs-4059R-A555) (Bioss Antibodies Inc., Woburn, MA, USA) diluted to 1/500 for 30 min. In addition, cells were incubated overnight at 4 °C with a mouse monoclonal antibody to human adiponectin (ab22554) (Abcam, Cambridge, UK) diluted 1/200 in PBS containing 1% BSA, and Alexa Fluor™ 488 goat anti-mouse IgG 1/1000 (A11029, Thermo Fisher Scientific Inc., Waltham, MA, USA) as secondary antibody for 1 h at room temperature. Finally, cell nuclei were counterstained with UltraCruz™ Mounting Medium for fluorescence with DAPI (Santa Cruz Biotechnology, Santa Cruz, CA, USA) and analyzed with an Olympus BX51 microscope equipped with an Olympus DP70 digital camera (Olympus, Glostrup, Denmark). Immunohistochemical techniques were performed in the Imaging platform of the Institute of Biomedical Research in Malaga (IBIMA). Quantification of oxLDL staining was performed with an ImageJ 1.50i program National Institute of Mental Health, Bethesda, MD, USA).

### 2.8. Oil Red-O Staining

HMSC from non-obese and morbidly obese subjects were differentiated into adipocytes as described above. HMSC and in vitro differentiated adipocytes were washed with PBS, incubated with oil red-O staining solution for 30 min and analyzed with an Olympus BX51 microscope equipped with an Olympus DP70 digital camera (Olympus, Glostrup, Denmark) [21]. In addition, stained lipid droplets from in vitro differentiated adipocytes were quantified as the absorbance at 520 nm after solubilization with 96% ethanol [22].

### 2.9. Cellular Cholesterol Quantification

HMSC from non-obese subjects were differentiated into adipocytes as described above. In vitro differentiated adipocytes were incubated with 0, 25, and 50 μg/mL of oxLDL for 24h. Afterwards, cells were washed twice with PBS, dissociated with trypsin from the well in which they were cultured, and centrifuged to pellet cells. Lipids were extracted by the addition of 1 mL chloroform/methanol (2:1) to the cell pellet after sample homogenization. The organic phase was withdrawn and evaporated under a current of nitrogen. The extracts were re-suspended in isopropanol, and the concentration of free (FC) and total cholesterol was determined by using commercial kits (FUJIFILM Wako Chemicals Europe GmbH, Neuss, Germany). Esterified cholesterol (EC) was calculated as total cholesterol—FC [23]. The concentration of total and FC per well was normalized by total cell protein concentration determined according to the bicinchoninic acid method (Thermo Fisher Scientific Inc. Rockford, IL, USA). Each treatment was performed in triplicate.

### 2.10. Glucose Uptake

HMSC from non-obese and morbidly obese subjects were differentiated into adipocytes as described above. Glucose uptake was determined in the in vitro differentiated adipocytes incubated with 0, 25, and 50 μg/mL of oxLDL for 24h using the 2-deoxyglucose method with the Glucose Uptake Colorimetric Assay Kit (ab136955, Abcam, Cambridge, MA, USA) according to the manufacturer’s instructions. Each treatment was performed in triplicate. 

### 2.11. Statistical Analysis

All analyses were performed using R statistical software, version 2.8.1 (Department of Statistics, University of Auckland, Auckland, New Zealand; http://www.r-project.org/). All the experiments were performed with a male/female ratio as close as 1/2 as possible, with no significant differences according to sex. Differences between two groups were compared by the Mann–Whitney test. Differences between two related variables were analyzed by the Wilcoxon test. Differences between the effects of MDA-LDL doses were made with a repeated-measure ANOVA. Values were statistically significant when *p* ≤ 0.05. The results are represented as the mean ± standard deviation (SD) in tables and as the mean ± standard error in figures.

## 3. Results

### 3.1. OxLDL Uptake by Visceral In Vitro Differentiated Adipocytes

Initially, after 15 days of differentiation, no significant differences were found in Oil Red-O staining between non-obese and morbidly obese subjects (Figure 1B). To establish whether oxLDL is uptaken by human visceral adipocytes, in vitro differentiated adipocytes from non-obese subjects (*n* = 4) and morbidly obese subjects (*n* = 4) were incubated with 50 μg/mL oxLDL. After 4 h of incubation at 37 °C, the oxLDL uptake by differentiated adipocytes was confirmed by immunofluorescence staining (Figure 1D). No significant differences were found in oxLDL staining (100% vs. 97 ± 6%, *p* = 0.526), cell shape or size of in vitro differentiated adipocytes between non-obese and morbidly obese subjects.

### 3.2. Esterified Cholesterol within Visceral In Vitro Differentiated Adipocytes Increases with OxLDL

**Figure 2:**
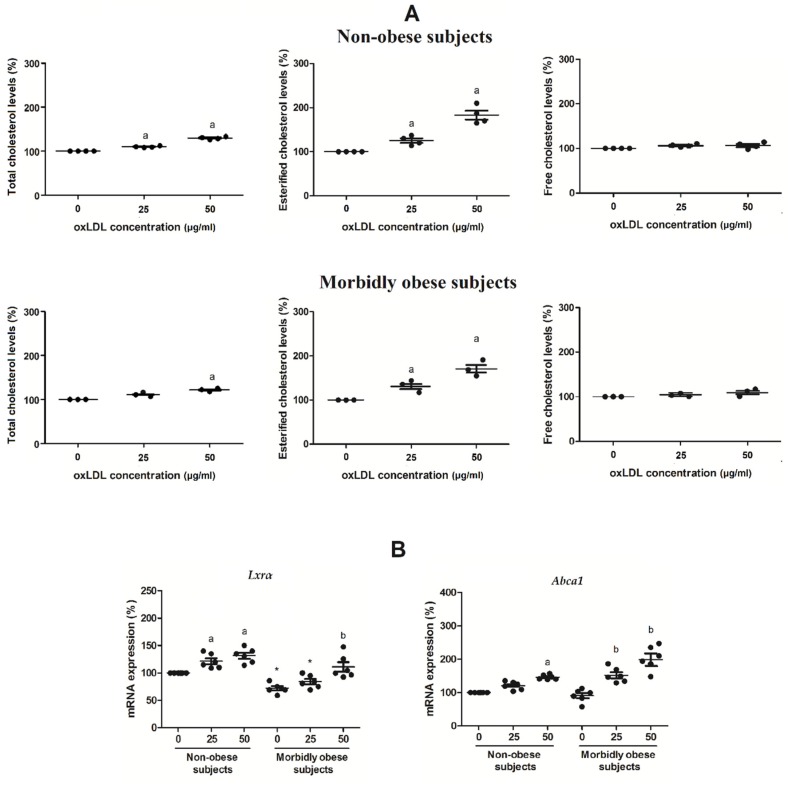
(**A**) Total cholesterol, esterified cholesterol and free cholesterol levels in human visceral in vitro differentiated adipocytes from non-obese (*n* = 4) and morbidly obese subjects (*n* = 3) incubated with different doses of oxLDL (0, 25, and 50 μg/mL oxLDL). ^a^ *p* < 0.05: significant differences with regard to 0 μg/mL oxLDL. (**B**) levels of mRNA expression of *Lxrα* and *Abca1* in human visceral in vitro differentiated adipocytes obtained from HMSC from non-obese (*n* = 6) and morbidly obese subjects (*n* = 6) incubated with 0, 25, and 50 μg/mL of oxLDL. ^a^ *p* < 0.05: significant differences with regard to 0 μg/mL oxLDL within non-obese subjects. ^b^ *p* < 0.05: significant differences with regard to 0 μg/mL oxLDL within morbidly obese subjects. * *p* < 0.05: significant differences for each dose between non-obese and morbidly obese subjects.

To corroborate whether oxLDL is uptaken by adipocytes, we measured the level of total cholesterol, FC and EC in human visceral in vitro differentiated adipocytes from non-obese (*n* = 4) and morbidly obese subjects (*n* = 3) incubated with 0, 25 and 50 μg/mL oxLDL for 24 h. The incubation with oxLDL led to a significant increase of total cholesterol and EC in adipocytes in a dose-dependent response with the increasing concentrations of oxLDL (*p* < 0.05) (Figure 2A). However, FC did not change significantly (Figure 2A). No significant differences were found in total cholesterol, FC, and EC between non-obese and morbidly obese subjects.

Since the incubation with oxLDL produces an increase of oxLDL uptake by visceral in vitro differentiated adipocytes and an increase of total cholesterol and EC, we wanted to check if there is a stimulation of the LXR pathway, which is stimulated by the presence of oxysterols. OxLDL increased *Lxrα* and *Abca1* expression in non-obese and in morbidly obese subjects (*p* < 0.05) (Figure 2B). In addition, we found a lower level of *Lxrα* in morbidly obese subjects than in non-obese subjects (Figure 2B). However, we did not find significant differences in *Abca1* expression between non-obese and morbidly obese subjects. 

### 3.3. OxLDL Produces an Increase in Insulin Resistance of Visceral In Vitro Differentiated Adipocytes

**Figure 3:**
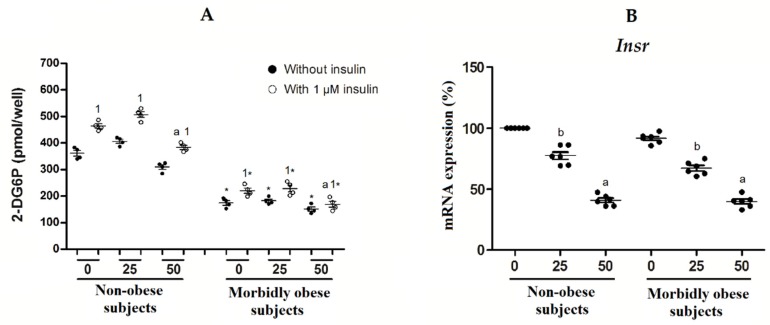
(**A**) Glucose uptake in human visceral in vitro differentiated adipocytes obtained from HMSC from non-obese (*n* = 4) and morbidly obese subjects (*n* = 4) incubated with 0, 25, and 50 μg/mL of oxLDL for 24 h. ^a^ *p* < 0.05: significant differences between 50 μg/mL with 0 and 25 μg/mL oxLDL. ^1^ *p* < 0.05: significant differences with regard to its control (non-insulin treated). * *p* < 0.05: significant differences for each dose between non-obese and morbidly obese subjects; (**B**) levels of mRNA expression of insulin receptor (*Insr*) in human visceral in vitro differentiated adipocytes obtained from HMSC from non-obese (*n* = 6) and morbidly obese subjects (*n* = 6) incubated with 0, 25, and 50 μg/mL of oxLDL for 24h. ^a^ *p* < 0.05: significant differences between 50 μg/mL with 0 and 25 μg/mL oxLDL. ^b^ *p* < 0.05: significant differences between 0 and 25 μg/mL oxLDL.

Furthermore, we wanted to analyze the involvement of oxLDL in the development of insulin resistance in adipocytes. First, we found that insulin-induced glucose uptake was significantly decreased with 50 μg/mL oxLDL with regard to 0 and 25 μg/mL oxLDL in both non-obese (*n* = 4) and morbidly obese subjects (*n* = 4) (Figure 3A). Second, morbidly obese subjects had lower glucose uptake than non-obese subjects both with or without insulin (*p* < 0.05). Third, the insulin-stimulated glucose uptake was significantly increased with regard to its control (non-insulin treated) in both non-obese and morbidly obese subjects (*p* < 0.05) (Figure 3A). Moreover, INSR expression shows a similar profile in both non-obese and morbidly obese subjects, without significant differences between them, and with a significant decrease with 25 and 50 μg/mL oxLDL (Figure 3B). 

### 3.4. OxLDL Modifies the Secretion of Adipocytokines

**Figure 4:**
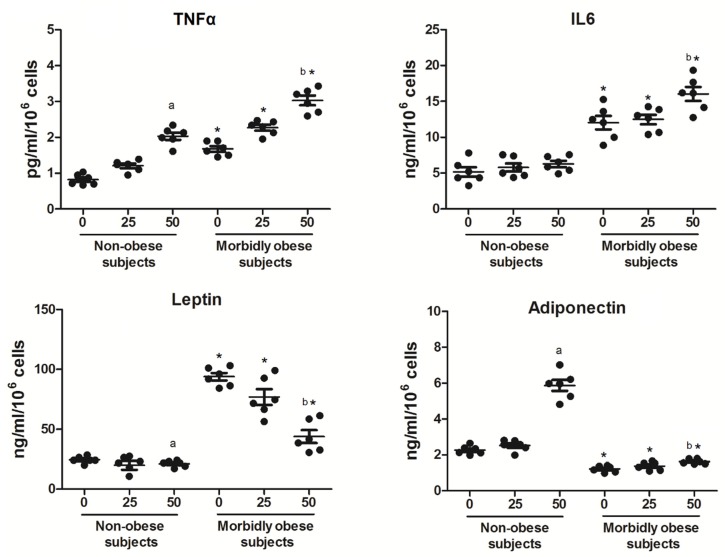
Levels of adipocitokines in the culture medium of in vitro differentiated adipocytes obtained from HMSC from non-obese (*n* = 6) and morbidly obese subjects (*n* = 6) incubated with 0, 25, and 50 μg/mL of oxLDL for 24h. ^a^ *p* < 0.05: significant differences with regard to 0 μg/mL oxLDL within non-obese subjects. ^b^ *p* < 0.05: significant differences with regard to 0 μg/mL oxLDL within morbidly obese subjects. * *p* < 0.05: significant differences for each dose between non-obese and morbidly obese subjects.

On the other hand, we wanted to check whether oxLDL could modify the levels of adipocytokines secreted by adipocytes. In addition, we wanted to determine whether the secretion of adipocytokines by adipocytes could be affected depending on the type of subjects (non-obese (*n* = 6) and morbidly obese subjects (*n* = 6)). As shown in Figure 4, human visceral in vitro differentiated adipocytes showed a significant increase of TNF-α secretion only with 50 μg/mL oxLDL, both in non-obese and morbidly obese subjects (*p* < 0.05). TNF-α secretion is also higher in differentiated adipocytes from morbidly obese subjects than from non-obese subjects (*p* < 0.05). With regard to IL-6 secretion, there was a significant increase only with 50 μg/mL oxLDL in differentiated adipocytes from morbidly obese subjects (*p* < 0.05). IL-6 secretion is also higher in differentiated adipocytes from morbidly obese subjects than from non-obese subjects (*p* < 0.05). With regard to leptin secretion, there was a significant decrease only with 50 μg/mL oxLDL, both in differentiated adipocytes from non-obese and morbidly obese subjects (*p* < 0.05). Leptin secretion is also higher in differentiated adipocytes from morbidly obese subjects than from non-obese subjects (*p* < 0.05). With regard to adiponectin secretion, there was a significant increase only with 50 μg/mL oxLDL, both in differentiated adipocytes from non-obese and morbidly obese subjects (*p* < 0.05), although much higher in non-obese subjects. Adiponectin secretion is lower in differentiated adipocytes from morbidly obese subjects than from non-obese subjects (*p* < 0.05).

### 3.5. OxLDL Increases the Expression of SRs in Visceral In Vitro Differentiated Adipocytes

**Figure 5:**
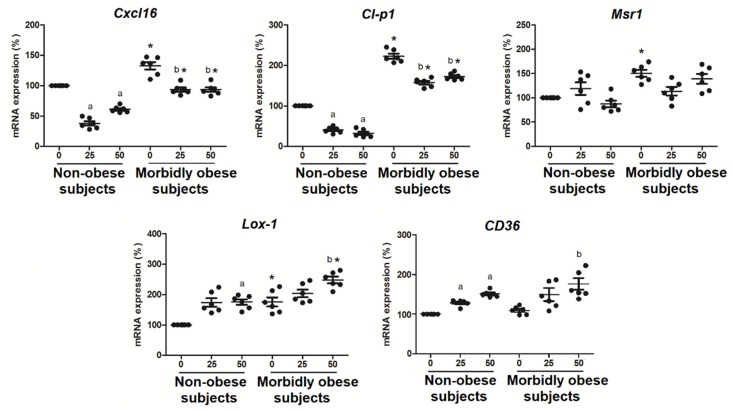
Levels of mRNA expression of scavenger receptors in the in vitro differentiated adipocytes obtained from HMSC from non-obese (*n* = 6) and morbidly obese subjects (*n* = 6) incubated with 0, 25 and 50 μg/mL of oxLDL for 24h. ^a^ *p* < 0.05: significant differences with regard to 0 μg/mL oxLDL within non-obese subjects. ^b^ *p* < 0.05: significant differences with regard to 0 μg/mL oxLDL within morbidly obese subjects. * *p* < 0.05: significant differences for each dose between non-obese and morbidly obese subjects.

Since oxLDL binds to human visceral in vitro differentiated adipocytes, first we wanted to check whether oxLDL produced an increase of their receptors. In addition, 25 and 50 μg/mL oxLDL significantly decreased *Cl-p1* (*p* < 0.05) and *Cxcl16* mRNA expression (*p* < 0.05) in non-obese (*n* = 6) and morbidly obese subjects (*n* = 6) (Figure 5). Furthermore, 50 μg/mL oxLDL significantly increased *Lox-1* (*p* < 0.05) and *CD36* mRNA expression in non-obese and morbidly obese subjects (Figure 5). In addition, *Cxcl16* (*p* < 0.05), *Cl-p1* (*p* < 0.05), and *Lox-1* (*p* < 0.05) had higher levels in adipocytes from morbidly obese subjects than from non-obese subjects.

**Figure 6:**
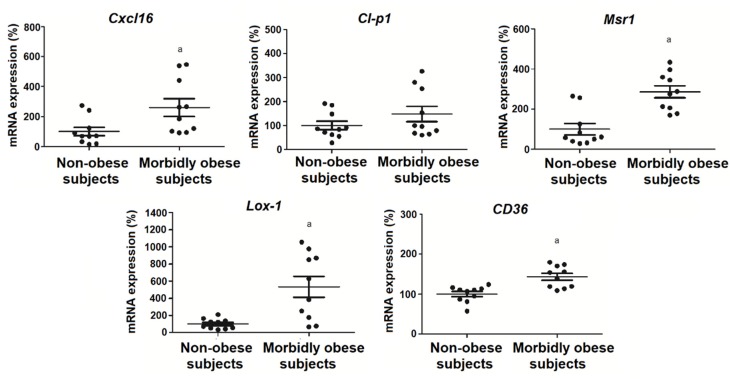
Levels of mRNA expression of scavenger receptors in visceral mature adipocytes obtained from visceral adipose tissue of non-obese (*n* = 10) and morbidly obese subjects (*n* = 10). ^a^ *p* < 0.05: significant differences with regard to non-obese subjects.

We also analyzed the expression of the same genes in visceral mature adipocytes from non-obese (*n* = 10) and morbidly obese subjects (*n* = 10) (Figure 6). We found an increase of *Cxcl16* (*p* < 0.05), *Msr1* (*p* < 0.05), *Lox-1* (*p* < 0.05), and *CD36* (*p* < 0.05) in morbidly obese subjects (Figure 6), which is a tendency also found in the in vitro differentiated adipocytes (Figure 5).

### 3.6. Oxldl Modifies the Expression of Apoptosis, Necrosis, and Autophagy Markers in Visceral In Vitro Differentiated Adipocytes

**Figure 7:**
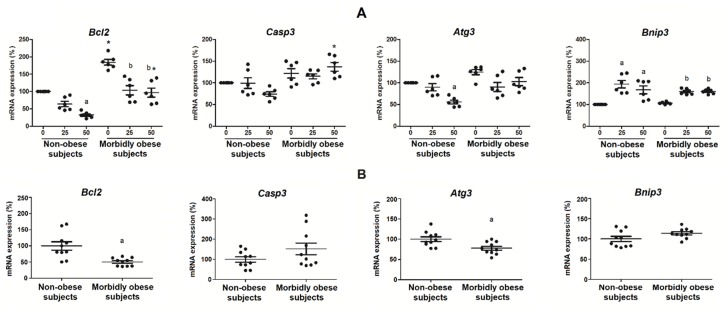
(**A**) Levels of mRNA expression of *Bcl2, Casp3, Atg3* and *Bnip3* in the in vitro differentiated adipocytes obtained from HMSC from non-obese (*n* = 6) and morbidly obese subjects (*n* = 6) incubated with 0, 25, and 50 μg/mL of oxLDL for 24h. ^a^ *p* < 0.05: significant differences with regard to 0 μg/mL oxLDL within non-obese subjects. ^b^ *p* < 0.05: significant differences with regard to 0 μg/mL oxLDL within morbidly obese subjects. * *p* < 0.05: significant differences for each dose between non-obese and morbidly obese subjects; (**B**) levels of mRNA expression *Bcl2, Casp3, Atg3* and *Bnip3* in visceral mature adipocytes obtained from visceral adipose tissue of non-obese (*n* = 10) and morbidly obese subjects (*n* = 10). ^a^ *p* < 0.05: significant differences with regard to non-obese subjects.

*Bcl2* mRNA expression, an anti-apoptotic protein, significantly decreased with the exposure to 50 μg/mL oxLDL in non-obese subjects (*p* < 0.05) (*n* = 6), and with the exposure to 25 and 50 μg/mL in morbidly obese subjects (*p* < 0.05) (*n* = 6) (Figure 7A). *Bnip3* mRNA expression, a protein involved in necrosis and apoptosis, significantly increased with 25 and 50 μg/mL of oxLDL in both non-obese (*p* < 0.05) and morbidly obese subjects (*p* < 0.05) (Figure 7A). *Atg3* mRNA expression, a protein involved in autophagocytosis, significantly decreased with the exposure to 50 μg/mL oxLDL in non-obese subjects (*p* = 0.002) (Figure 7A). *Casp3* mRNA expression, a pro-apoptotic protein, did not change significantly (Figure 7A). In addition, *Bcl2* (*p* < 0.05) had higher levels in adipocytes from morbidly obese subjects than from non-obese subjects.

We also analyzed the expression of the same genes in visceral mature adipocytes from non-obese (*n* = 10) and morbidly obese subjects (*n* = 10) (Figure 7B). We found a decrease of *Bcl2* (*p* < 0.05) and *Atg3* (*p* < 0.05) and a slight increase, but not significant, of *Casp3* and *Bnip3* in morbidly obese subjects.

### 3.7. OxLDL Modifies the Expression of Nrf2 and NF-kB in Visceral In Vitro Differentiated Adipocytes

**Figure 8:**
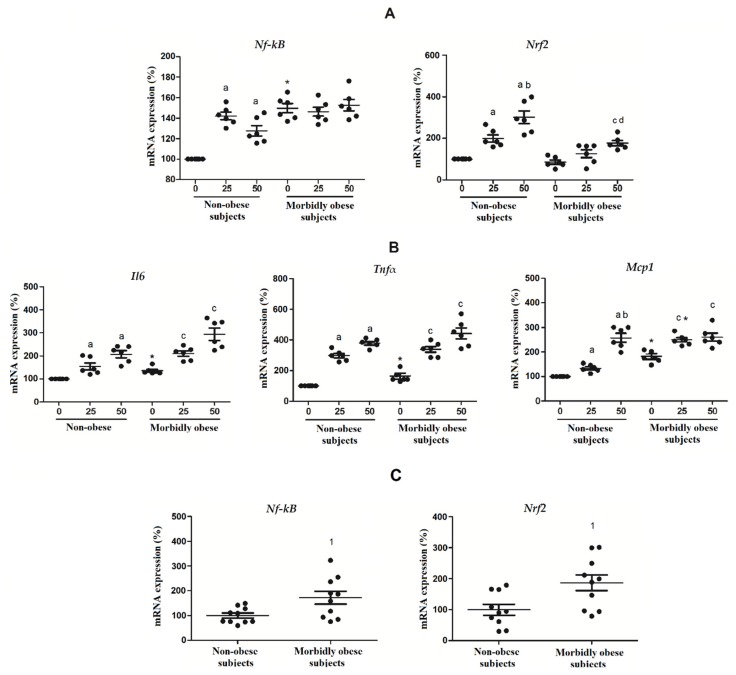
(**A**) Levels of mRNA expression of *Nf-kB* and *Nrf2* and (**B**) NF-κB-target genes (*Tnfα, Il6* and *Mcp1*) in the in vitro differentiated adipocytes obtained from HMSC from non-obese (*n* = 6) and morbidly obese subjects (*n* = 6) incubated with 0, 25 and 50 μg/mL of oxLDL for 24h. ^a^ *p* < 0.05: significant differences with regard to 0 μg/mL oxLDL within non-obese subjects. ^b^ *p* < 0.05: significant differences with regard to 25 μg/mL oxLDL within non-obese subjects. ^c^ *p* < 0.05: significant differences with regard to 0 μg/mL oxLDL within morbidly obese subjects. ^d^ *p* < 0.05: significant differences with regard to 25 μg/mL oxLDL within morbidly obese subjects. * *p* < 0.05: significant differences for each dose between non-obese and morbidly obese subjects; (**C**) levels of mRNA expression of *Nf-kB* and *Nrf2* in visceral mature adipocytes obtained from visceral adipose tissue of non-obese (*n* = 10) and morbidly obese subjects (*n* = 10). ^1^ *p* < 0.05: significant differences with regard to non-obese subjects.

*Nrf2* and *Nf-κB* mRNA expressions, two transcription factors activated by oxLDL which are involved in the inflammatory gene expression and in cell cycle regulation, were also analyzed. *Nf-κB* significantly increased with 25 and 50 μg/mL of oxLDL in non-obese subjects (*p* < 0.05) (Figure 8A). *Nrf2* significantly increased with the exposure to 25 and 50 μg/mL oxLDL in non-subjects (*p* < 0.05) and with 50 μg/mL of oxLDL in morbidly obese subjects (*p* < 0.05) (Figure 8A). We also analyzed the expression of three NF-κB-target genes, such as *Tnfα, Il6,* and *Mcp1* (Figure 8B). We found that the expression of these three genes was increased similarly to the expression of *Nf-κB*. 

In visceral mature adipocytes (Figure 8C), we found an increase of *Nf-κB* (*p* < 0.05) and *Nrf2* (*p* < 0.05) in morbidly obese subjects.

## 4. Discussion

In this study, we showed that oxLDL is uptaken by visceral in vitro differentiated adipocytes increasing the esterified cholesterol levels and decreasing insulin-induced glucose uptake. The exposure of visceral in vitro differentiated adipocytes to high oxLDL concentrations (50 μg/mL) produced a change in SR expression (*Lox-1, Cl-p1* and *Cxcl16*) and in adipocytokine secretion (TNF-α, IL-6, leptin, and adiponectin), both in non-obese and in morbidly obese subjects. In addition, oxLDL produced a change in apoptosis and autophagy markers (*Bcl2, Atg3* and *Bnip3*).

### 4.1. Effects of oxLDL on Intracellular Cholesterol and Insulin Resistance

The relation between oxLDL with obesity and insulin resistance has been analyzed in different studies with contradictory results [24,25,26,27]. To clarify this relation, we performed different in vitro experiments. We found that high oxLDL levels produced changes in the metabolism of visceral adipocytes, which are very involved in the regulation of obesity and insulin resistance. First, in this study, we found that human adipocytes may uptake oxLDL and increase their EC levels in a dose-dependent manner, as is also found in 3T3-L1 adipocytes [2] or in animal adipocytes [28,29]. This increase of intracellular cholesterol would be reflected mainly in the levels of EC because LDL is the lipoprotein with more content in EC [30]. However, we do not know the cause of the significant increase found in EC and not in FC. Part of FC from oxLDL could be transformed into EC by a mechanism not considered in this study [29]. In addition, the increase of *Abca1* expression may be partly involved. The excess of FC could be effluxed to apoA-I via ABCA1, forming nascent HDL particles [31,32]. This increase of intracellular cholesterol overload may be a mechanism for the increase of endoplasmic-reticulum stress by oxLDL. On the other hand, oxLDL is a source of oxysterols, which stimulates LXRα. As expected, we have found that oxLDL produced a stimulation of *Lxrα* and the subsequent induction of LXR-target genes such as *Abca1*. ABCA1 has been reported to be a key regulator of adipocyte lipogenesis and lipid accretion [10]. Second, a high dose of oxLDL decreased the insulin-induced glucose uptake together with a decrease in *Insr* expression in both non-obese and morbidly obese subjects. Although the non-obese group could have a slight insulin resistance, the non-obese and morbidly obese subjects from whom the samples came had no significant differences in HOMA-IR, and the effects of oxLDL were similar in *Insr* expression. In the regulation of insulin resistance, NF-κB and Nrf2 could be involved [33,34,35,36]. However, our results do not fully explain the regulation of insulin resistance by oxLDL through NF-κB and Nrf2. In agreement with our findings, Scazzicchio et al. provided findings that oxLDL induced insulin resistance by impairing insulin signals at multiple levels in 3T3-L1 adipocytes [33]. However, most of these studies were performed on animal models or cell lines, not in human adipocytes as in our study. 

### 4.2. Effects of OxLDL on Adipocytokine Secretion

To analyze the possible effect of oxLDL on adipocytokines, we performed an in vitro incubation with oxLDL. We found that high doses of oxLDL produced a decrease in leptin, as a previous study also shows in 3T3-L1 adipocytes [37], and an increase in adiponectin secretion. This effect on adiponectin could probably be mediated by peroxisome proliferator activated receptor gamma (PPARγ). This transcription factor, which is expressed mainly in adipose tissue, is stimulated by oxLDL [38] and is a positive regulator of adiponectin gene expression and secretion [39]. These results could be relevant since leptin and adiponectin have emerged as modulators of the immune system [40]. The change in its secretion could be counteracting the increase of the proinflammatory cytokine secretion produced by oxLDL. Leptin has proinflammatory effects, increasing neutrophil recruitment, macrophages and NK cell activation, lymphocytes chemotaxis, and T cell activation, and decreasing Treg recruitment [41]. Meanwhile, adiponectin has a role as an anti-inflammatory molecule reducing T cell responsiveness, B cell lymphopoiesis, monocyte adhesion, toll like receptor 4 (TLR4) activation, and proinflammatory mediators such as TNF-α while increasing the production of IL-10 [42]. In spite of these in vitro effects, the in vivo situation is different, with serum oxLDL showing a positive correlation with leptin and negative with adiponectin [42,43].

OxLDL is associated with atherosclerosis, insulin resistance, and metabolic syndrome [3,4,5]. In these pathologies, there is a chronic low-grade inflammation in which adipocytes may be involved through different adipocytokines. We found that high oxLDL levels increased the secretion of proinflammatory adipocytokines (TNF-α and IL6), as in a previous study performed on macrophages [44]. This increase could be partially mediated by the increase found in *Nf-κB* expression, since they are specific NF-κB target genes [45]. These results may help to explain the link between oxLDL and systemic inflammation. However, other studies performed in macrophages show different results [46,47]. These different findings could be due to the different function and metabolic regulation of these cells. The regulation of adipocytokine secretion by oxLDL follows the same trend in non-obese as in morbidly obese subjects. However, adipocytokine secretion is strongly influenced by obesity, with higher TNF-α, IL-6 and leptin and lower adiponectin levels in adipocytes from morbidly obese subjects than from non-obese subjects.

### 4.3. Effects of OxLDL on Scavenger Receptors

OxLDL may be also involved in the regulation of SR expression. In a previous study carried out by our group, we found SR expression in human visceral in vitro differentiated adipocytes [48]. As previous studies in other types of cells [9,49], our results show that oxLDL can upregulate their own receptors (*Lox-1* and *CD36*) in a concentration dependent fashion. This could have important repercussions, since LOX-1 and CD36 are involved in proinflammatory signaling and atherogenesis in other types of cells [50]. This increase of LOX-1 could sensitize the adipocytes to uptake more oxLDL and, in this way, increase the proinflammatory potential of adipocytes and contribute to the low-grade inflammation present in obesity. These results may help understand the link between oxLDL and systemic inflammation. However, oxLDL produced a significant decrease in *Cxcl16*, another type of SR. This may have different implications. CXCL16 effects depend on cell type and on whether CXCL16 is soluble or membrane-bound [51]. Membrane-bound CXCL16 may be a receptor for oxidized LDL or an adhesion protein for CXCR6-expressing cells. Additionally, soluble CXCL16 functions as a classical chemo-attractant for cells expressing CXCR6, and may be involved in proinflammatory gene transcription, matrix metalloproteinase activity, increased cell-cell adhesion, etc. [51]. In the regulation of SR levels, proprotein convertase subtilisin/kexin type 9 (PCSK9) may also be of interest for its involvement in the degradation of certain SRs [52,53].

### 4.4. Effects of OxLDL on Apoptosis, Necrosis, and Autophagy Markers

On the other hand, oxLDL induces apoptosis [13]. In our study, we found that *Casp3* expression did not change, although *Bcl2* expression decreased. In agreement with our findings, there are studies that have shown that oxLDL reduces the expression of the antiapoptotic proteins BCL2 through a LOX-1 receptor mediated pathway promoting susceptibility to apoptosis [54,55]. Downregulation of BCL2 promotes apoptotic cell death [54]. This decrease of *Bcl2* expression could also promote autophagy and aggravate the injury of adipocytes [56]. This effect occurs via the overexpression of *Lox-1*, which is also found in our study, and the subsequent decrease of protective autophagic response [57]. This scenario, a higher *Lox-1* and a lower *Bcl2* expression, is also found in mature adipocytes, as shown in a previous study in human adipose tissue [58]. 

However, other studies also show that oxLDL causes cell death by mechanisms other than apoptosis [59]. We found that oxLDL produces an increase of *Bnip3*, independent of oxLDL dose and type of subject. BNIP3 has proapoptotic functions [60], and has also been implicated in necrosis, autophagic cell death, and mitophagy [61]. BNIP3 regulates mitochondrial metabolism by removing damaged mitochondria via autophagy/mitophagy, and protects cells from death [62]. However, its role in visceral adipocytes, a mitochondria-poor cell, has been hardly evaluated. Our results suggest that BNIP3 could play a role in the regulation of adipocyte survival. 

### 4.5. Effects of OxLDL on Transcription Factors

Many biological effects of oxLDL are mediated through different transcription factors, such as NF-kB and Nrf2 [38], being interrelated between them [63]. We found that oxLDL increased *Nf-kB* and three of the most highly induced NF-κB-dependent genes [45,64], such as *Tnfα*, *Il6*, and *Mcp1*. We found that oxLDL increased *Nf-κB* expression in non-obese subjects, since, in morbidly obese subjects, it was increased in any experimental condition, independently of the oxLDL dose. This agrees with the increase found in visceral mature adipocytes in morbidly obese subjects with regard to non-obese subjects. Regarding *Nrf2*, oxLDL produced an increase of *Nrf2* in human visceral in vitro differentiated adipocytes, as in other studies [9,65,66]. Our results also agree, although in the opposite direction, with the increased expression of *Cxcl16* found in mice Nrf2^-/-^ peritoneal macrophages [67]. Although in this study, MSR1 and LOX-1 were also increased [67], our results did not show this association, suggesting that the expression of *Msr1, Lox-1* and *Cl-p1* in adipocytes could be regulated by oxLDL through other factors different to *Nrf2*. We also found an increase of *Nrf2* in visceral mature adipocytes from morbidly obese subjects. This increase could be compensating for the increase in oxidative stress present in these subjects [68], since Nrf2 is a regulator of the antioxidant reactions and plays an important role against inflammation as well as oxidative stress [69]. 

This model of human mesenchymal stem cell-derived adipocytes is interesting. In general, the effects of increased an oxLDL dose follow the same trend, both in non-obese and morbidly obese subjects. However, our results agree with a previous study in subcutaneous adipocytes showing that obesity determines the phenotypic profile and functional characteristics of human HMSC from adipose tissue [18], although there are slight differences in the type of cells and the culture media used. The results of that study and those shown here suggest that in vitro differentiated human visceral adipocytes from morbidly obese subjects may be retaining certain characteristics of the HMSC from which they come. Morbidly obese subjects have increased levels of leptin and inflammatory markers [70,71], decreased levels of adiponectin [72], an increase of insulin resistance [71], and an increased level of SR expression in mature adipocytes. Some of these findings are also found in differentiated human visceral adipocytes from morbidly obese subjects: an increase of adipocytokine secretion (or decrease for adiponectin), a slight increase of SR expression, a similar profile of transcription factors, and a lower glucose uptake. We also found that the expression of some genes is not consistent between in vitro differentiated human visceral and mature adipocytes. This could be the consequence of the different conditions in which these cells have developed, i.e., the state of low-grade chronic inflammation associated with obesity or the different hormone levels between in vitro and in vivo conditions. However, our study has some limitations. Due to the low mRNA expression of some receptors, analyses of protein levels, phosphorylated AKT protein levels, as an indicator of insulin resistance, and cleaved-caspase-3 could not be performed as the adipocytes were used for mRNA isolation, and not for protein isolation.

## 5. Conclusions

In conclusion, oxLDL could sensitize adipocytes to a lower insulin-induced glucose uptake and a more proinflammatory phenotype, which could be involved in the chronic low-grade inflammation present in pathologies in which oxLDL is increased. Moreover, oxLDL can also modify the expression of their receptors, mainly in LOX-1, which could lead to a possible amplification of proinflammatory and proapoptotic signaling of oxLDL. This alteration of adipocyte metabolism could be closely linked to the development of T2DM and atherosclerosis. However, more studies are needed to analyze in detail different pathways not analyzed in this study, by overexpression or inhibition of certain SRs or other transcription factors involved in the response to oxLDL. In addition, we have been able to show how cells can retain part of the characteristics of the subjects they come from.

## Figures and Tables

**Table 1:** Anthropometric and biochemical variables of the non-obese and morbidly obese subjects included in this study.

Variables	Non-Obese Subjects (*n* = 10)	Morbidly Obese Subjects (*n* = 10)
Sex (male/female)	4/6	4/6
Age (years)	42.18 ± 15.1	40.79 ± 9.02
Weight (Kg)	74.3 ± 12.5	131.8 ± 20.1 ^¶^
BMI (kg/m^2^)	27.0 ± 3.4	49.3 ± 7.8 ^¶^
Waist (cm)	98.2 ± 11.3	135.5 ± 15.7 ^¶^
Hip (cm)	101.0 ± 9.1	149.1 ± 15.3 ^¶^
Waist/hip ratio	0.97 ± 0.12	0.91 ± 0.10
SBP	127.4 ± 19.7	149.1 ± 15.3 ^∗^
DBP	80.6 ± 10.2	82.7 ± 12.5
Glucose (mg/dL)	96.7 ± 17.5	91.3 ± 16.1
Cholesterol (mg/dL)	201.1 ± 35.3	185.0 ± 37.7
Triglycerides (mg/dL)	135.3 ± 61.1	116.1 ± 51.7
HDL (mg/dL)	48.5 ± 8.5	48.8 ± 11.2
LDL (mg/dL)	126.8 ± 32.4	113.6 ± 31.9
Insulin (μIU/mL)	9.2 ± 6.3	13.6 ± 4.2
HOMA-IR	2.7 ± 1.6	4.4 ± 3.5
Oxidized-LDL (mU/L)	60562.5 ± 10687.7	70658.1 ± 13975.4 ^∗^

The results are given as the mean ± standard deviation. BMI: body mass index. SBP: systolic blood pressure. DBP: diastolic blood pressure. HDL: high density lipoproteins. LDL: low density lipoprotein. HOMA-IR: homeostasis model assessment of insulin resistance index. Significant differences between non-obese and morbidly obese subjects (^∗^ *p* < 0.05; ^¶^ *p* < 0.001).
